# Functionalizing Dendrimers for Targeted Delivery of Bioactive Molecules to Macrophages: A Potential Treatment for *Mycobacterium tuberculosis* Infection—A Review

**DOI:** 10.3390/ph16101428

**Published:** 2023-10-09

**Authors:** Claudia Sanhueza, Daniela Vergara, Catalina Chávez-Aravena, Felipe Gálvez-Jiron, Emigdio Chavez-Angel, Alejandro Castro-Alvarez

**Affiliations:** 1 Centro de Excelencia en Medicina Traslacional (CEMT), Facultad de Medicina, Universidad de La Frontera, Temuco 4811230, Chile; 2 Departamento de Ciencias Preclínicas, Facultad de Medicina, Universidad de La Frontera, Temuco 4811230, Chile; 3 Doctorado en Ciencias Mención Biología Celular y Molecular Aplicada, Facultad de Medicina, Universidad de La Frontera, Temuco 4811230, Chile; 4 Catalan Institute of Nanoscience and Nanotechnology (ICN2), CSIC and BIST, Campus UAB, Bellaterra, 08193 Barcelona, Spain

**Keywords:** tuberculosis, *Mycobacterium tuberculosis*, miRNA, dendrimers, host directed therapy

## Abstract

Tuberculosis (TB) is an infectious disease caused by *Mycobacterium tuberculosis* that replicates inside human alveolar macrophages. This disease causes significant morbidity and mortality throughout the world. According to the World Health Organization 1.4 million people died of this disease in 2021. This indicates that despite the progress of modern medicine, improvements in diagnostics, and the development of drug susceptibility tests, TB remains a global threat to public health. In this sense, host-directed therapy may provide a new approach to the cure of TB, and the expression of miRNAs has been correlated with a change in the concentration of various inflammatory mediators whose concentrations are responsible for the pathophysiology of *M. tuberculosis* infection. Thus, the administration of miRNAs may help to modulate the immune response of organisms. However, direct administration of miRNAs, without adequate encapsulation, exposes nucleic acids to the activity of cytosolic nucleases, limiting their application. Dendrimers are a family of highly branched molecules with a well-defined architecture and a branched conformation which gives rise to cavities that facilitate physical immobilization, and functional groups that allow chemical interaction with molecules of interest. Additionally, dendrimers can be easily functionalized to target different cells, macrophages among them. In this sense, various studies have proposed the use of different cell receptors as target molecules to aim dendrimers at macrophages and thus release drugs or nucleic acids in the cell of interest. Based on the considerations, the primary objective of this review is to comprehensively explore the potential of functionalized dendrimers as delivery vectors for miRNAs and other therapeutic agents into macrophages. This work aims to provide insights into the use of functionalized dendrimers as an innovative approach for TB treatment, focusing on their ability to target and deliver therapeutic cargo to macrophages.

## 1. Introduction

Tuberculosis (TB) is a disease of the lungs caused by *Mycobacterium tuberculosis*, which can result in severe morbidity and mortality [1]. According to World Health Organization (WHO) figures, 1.4 million people died of TB in 2021. According to the Global Tuberculosis Report 2022 [2], most of the estimated increase in TB deaths globally was accounted for by four countries: India, Indonesia, Myanmar, and the Philippines. However, if we look at the proportion of global cases, India had the highest number of cases, accounting for 26% of global cases in 2021.

At the moment, bacille Calmette–Guérin (BCG) is the only licensed TB vaccine. However, it has limited efficacy against pulmonary TB disease development because it does not prevent primary infection or reactivation of latent lung infection, which is the main source of the spread of the bacillus in the community. The effect of BCG vaccines on the transmission of *M. tuberculosis* is therefore limited [3].

On the other hand, although antibiotic treatment of drug-susceptible *M. tuberculosis* is generally effective, drug-resistant TB has a treatment efficacy below 50% and can, in a proportion of cases, develop into progressive, untreatable disease [4].

Thus, despite the progress of modern medicine, improvements in diagnosis and the development of drug susceptibility tests, TB remains one of the most threatening curable infectious diseases. Therefore, it is necessary to study new therapies for the treatment of TB.

**Figure 1:**
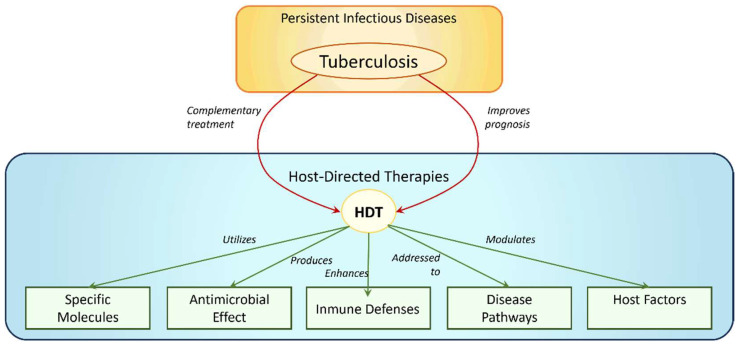
Schematic Representation of Diverse Host-Directed Therapies (HDT) in Tuberculosis (TB) Treatment.

In this sense, host-directed therapies (HDT) have appeared as complementary treatments for persistent infectious diseases such as TB. HDT is a treatment that uses specific molecules that produce an antimicrobial or beneficial effect by: (a) interfering with the host mechanisms used by the pathogen to persist or replicate in host tissues; (b) boosting the host’s immune defenses against the pathogen; (c) targeting pathways that may contribute to disease or immunopathology; (d) modulating host factors locally that are associated with pathogenic responses (Figure 1). Therefore, the use of HDT is intended to improve the prognosis of TB through immunomodulation [5].

Furthermore, the therapeutic modulation of immunity through cytokines is a way of supporting host defenses. Cytokines play a crucial role in immune cell function and, in theory, may serve as promising candidates for inclusion in complementary immunotherapies [6]. Thus, reducing excessive cytokine responses appears to be a promising HDT strategy for people with TB.

TB is a chronic granulomatous infectious disease caused by *M. tuberculosis* in humans. TB usually attacks the lungs, but it can affect any part of the body [7]. Infection occurs via aerosol and inhalation of a few droplets containing *M. tuberculosis* bacilli [8]. After infection, *M. tuberculosis* pathogenesis occurs in two stages. The first is an asymptomatic state that can persist for many years in the host, called latent TB. People with latent TB cannot spread the infection to other people [7].

However, when the tubercle bacilli overcome the immune system and multiply in the host, latent TB progresses to active TB. People with active TB are usually infectious and can expel the bacteria into the air, for example by coughing, and thereby infect other people [8].

Macrophages are responsible for the activation of protective immune responses to control or eliminate infection. Macrophages are widely distributed and strategically placed in many tissues of the body not only to combat infection but also to perform a vast array of other immunological, physiological and homeostatic functions, and therefore play important roles in disease control and progression [9]. Autophagy, carried out by macrophages, is a lysosomal degradative process that participates in cellular homeostasis by enabling the removal of defective organelles, protein aggregates, or intracellular microorganisms [10].

Macrophages are the major host of *M. tuberculosis* in humans. *M. tuberculosis* has evolved some strategies to counter autophagy defense and prompts the host to elicit an immune response that favors its persistence [4,11,12]. Several proteins produced by *M. tuberculosis* have been shown to modulate or inhibit macrophage autophagy and antimicrobial responses, enhancing the intracellular survival of *M. tuberculosis* in macrophages by scavenging cellular reactive oxygen species and upregulating IL-10 [13].

### 1.1. TB Current Therapies

Active TB disease can be treated by taking several drugs for 6 to 9 months. Currently, there are 10 drugs approved by the U.S. Food and Drug Administration (FDA) for treating TB. Of the approved drugs, the first-line anti-TB agents that form the core of treatment regimens are isoniazid, rifampin, ethambutol, and pyrazinamide [8,14]. However, the number of cases with isoniazid- and rifampin- resistant *M. tuberculosis*, the two most potent antituberculosis drugs, has increased in the last few years. In 2013, 480,000 new cases of multidrug-resistant tuberculosis (MDR-TB) were reported in the world, and in 2018 around half a million new cases of rifampicin-resistant TB were reported [15,16]. About 50% of reported MDR-TB cases culminate in the death of the patient. According to the Global Tuberculosis Report 2022, globally in 2021, 7.3% of people with rifampicin-resistant TB (RR-TB) had extensively drug-resistant TB (XDR-TB), which is resistant to rifampicin and isoniazid, as well as to any fluoroquinolone and at least one of three injectable second-line drugs (amikacin, capreomycin, or kanamycin). In total, there were an estimated 465,000 cases of rifampicin-resistant TB (RR-TB) in 2021, of which 39% were reported to have been tested for XDR-TB [2].

To address the challenges of TB, the World Health Organization (WHO) has released the “Strategy to End TB”, indicating that the target for 2050 is to have less than one TB patient per million people each year [17].

Thus, to overcome the global public health crisis of MDR-TB, several studies have been conducted on gene therapy and immunotherapy in recent years, and the results from these studies, at least in animal models, have been promising [17,18]. In this sense, different studies have indicated that miRNAs may regulate host–pathogen interactions in bacterial and viral infections, including *M. tuberculosis* infection [13].

### 1.2. miRNA Therapies

Gene therapy is a novel and promising tool for the treatment of many severe diseases, and the silencing of proteins using gene therapy is the safest and most efficient tool to treat diseases because it does not induce changes in the human genome [19].

**Figure 2:**
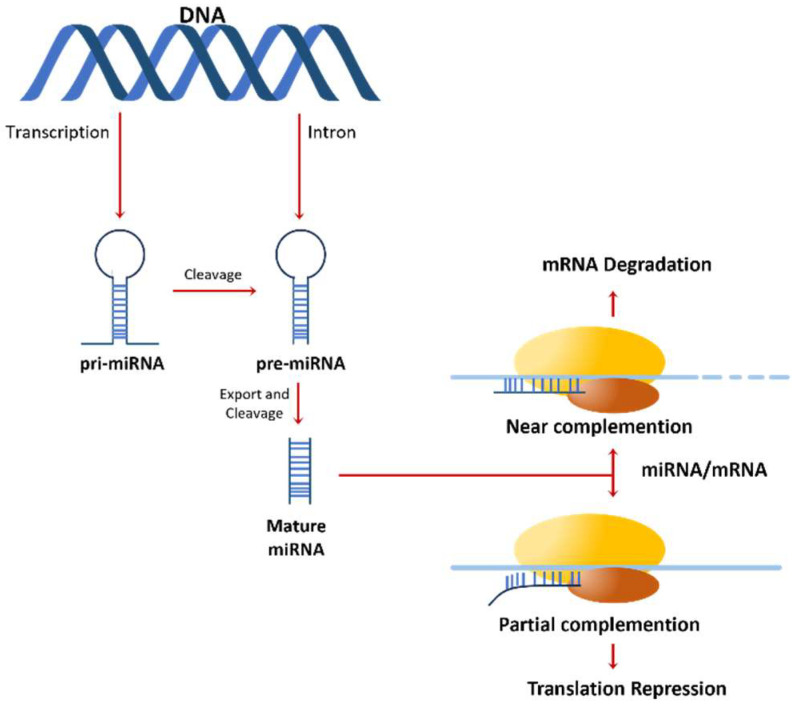
Illustration of the miRNA action mechanism from transcription: miRNA genes undergo transcription via RNA polymerase II, resulting in the formation of primary miRNA transcripts (pri-miRNAs). Subsequently, these pri-miRNAs are processed within the nucleus by RNase III enzymes, specifically Drosha and Dicer, leading to the formation of precursor miRNAs (pre-miRNAs). The Dicer enzyme further refines pre-miRNAs into mature miRNA duplexes. Adapted from Ryan et al. [22].

In this context, miRNAs are small RNA molecules that are typically 21–23 nucleotides in length. They are transcribed from endogenous genes and are processed by several key proteins to form mature miRNAs. Mature miRNAs can then bind to messenger RNA (mRNA) transcripts, leading to either mRNA degradation or translational repression (Figure 2). This process allows miRNAs to regulate gene expression at the post-transcriptional level and play a critical role in various biological processes, including development, differentiation, and homeostasis [20].

Remarkably, a single miRNA can regulate the expression of multiple mRNAs, thereby modulating entire gene networks and pathways. This phenomenon is known as miRNA-mediated gene regulation and highlights the versatility and specificity of miRNAs in controlling gene expression. Moreover, an mRNA transcript can be targeted by many different miRNAs, which can bind to various miRNA-binding sites within the mRNA molecule. The presence of multiple miRNA-binding sites within an mRNA transcript allows for a complex and precise regulation of gene expression at the post-transcriptional level [21].

Furthermore, miRNAs have been shown to play a key role in the immune response against infectious diseases. For example, during viral infections, miRNAs can modulate the expression of viral genes to either suppress viral replication or facilitate the host immune response. Similarly, miRNAs can regulate the expression of genes involved in host–pathogen interactions, such as genes encoding cytokines and chemokines, to modulate the host immune response and inflammation [23].

Many studies have characterized the function and regulatory mechanisms of miRNAs in the intracellular and extracellular organelles, as well as their role in human diseases, serving as a potential diagnostic and therapeutic target [13], and several miRNAs have been identified as regulators that play important roles to up-regulate or down-regulate innate immune response in the regulation of *M. tuberculosis* replication and infection [24]. The dysregulation of miRNA expression has also been implicated in TB pathogenesis, with some miRNAs promoting bacterial replication and others enhancing host defense mechanisms. This has led to the suggestion that miRNAs could be used as diagnostic biomarkers or therapeutic targets for TB treatments [25].

The activation of innate immune cells is regulated by various miRNAs, among them miRNA-155, miRNA-145, miRNA-146a, miRNA-21, and miRNA-26b [20]. These miRNAs play a key role in controlling both innate and adaptive immune responses against *M. tuberculosis* by targeting IFN-γ, which has been suggested as a potential biomarker for pulmonary TB due to its association with the clinical manifestation of the disease. A recent study by Li et al. (2018) [24], found that miRNA-26b modulates inflammatory cytokine expression, leading to the attenuation of the immune response. Specifically, overexpression of miRNA-26b resulted in a reduction of inflammatory cytokine secretion in *M. tuberculosis* infection such as IFN-γ, IL-1β, IL-6 and TNF-α [24]. Notably, IL-6 has been shown to be crucial in the induction of protective memory response [26], and the absence of IFN-γ has been linked to reduced phagocytosis in macrophages [27].

Direct delivery of miRNA is considered a promising method to boost the immune response against TB by targeting specific miRNAs that are downregulated during infection, such as miRNA-155 [20]. MiRNA-155 has a protective role against mycobacterial infections and its expression is induced in macrophages upon infection with mycobacteria. It acts as a positive regulator of TLR signaling, inducing the production of type I IFN, which is crucial for the immune response against TB [20]. Overexpression of miR-155 has been shown to promote autophagy and the maturation of mycobacterial phagosomes in macrophages, which facilitate the elimination of intracellular mycobacteria [28].

However, the efficacy of miRNA therapy is limited by poor targeting ability, short circulation time and the off-target effects of naked miRNA-based agents [29]. Additionally, miRNAs are highly hydrophilic and therefore unable to penetrate lipid cell membranes, and can be degraded by nucleases in the blood [30]. Therefore, the successful delivery of miRNA into the target cell should be achieved with the lowest toxicity and should provide shielding, targeting, and cellular uptake [29]. In order to overcome these barriers, different miRNA-loaded encapsulation platforms have been proposed including nanoparticles, liposomes, and dendrimers [29,30,31]. Dendrimers have garnered attention due to their unique physicochemical (such as solubility, specificity, stability, biodistribution and therapeutic efficiency), biological (e.g., ability to overcome issues to reach the right target(s) through first-pass effect, immune clearance, cell penetration, off-target interactions), and mechanical properties [32]. In addition, improved pharmacokinetic and pharmacodynamic behaviors demonstrate their strong potential in medicine as nanocarriers [33].

## 2. Dendrimers as Carriers

**Figure 3:**
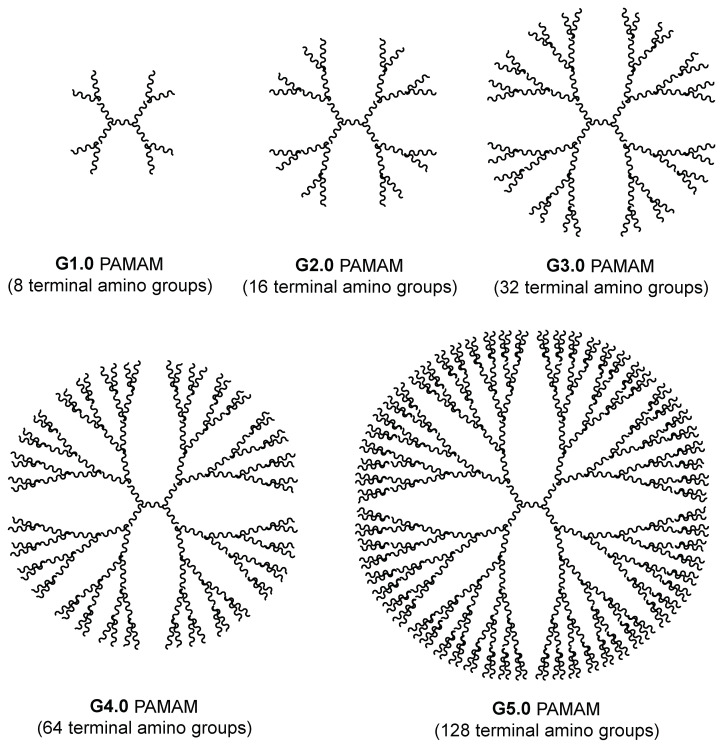
The 2D structure of PAMAM dendrimers at different generations.

Dendrimers are well-defined artificial polymers and consist of a central core molecule that acts as the root from which a number of highly branched, tree-like arms sprout in an ordered and symmetrical fashion (Figure 3) [31,32,33,34]. Dendrimers possess several functional groups responsible for high solubility and reactivity, and have empty internal cavities, making them suitable to act as carriers for drugs and nucleic acids [35]. The high water-solubility of cationic dendrimers positions them as efficacious nanocarriers for nucleic acids in biological applications [36]. Nucleic acids can be loaded by surface absorption or interior encapsulation to avoid degradation and immune responses [37].

Dendrimer synthesis offers the possibility of generating monodisperse macromolecular structures. Generally, they are prepared using a divergent or a convergent method. In both methods, the dendrimer grows outward from a multifunctional core molecule. First, the core molecule reacts with monomer molecules containing one reactive and two inactive groups, creating the first generation dendrimer. Then, the new periphery of the molecule is activated for reactions with more monomers to form new generations of dendrimers [38]. The voids inside dendrimers can accommodate ions and molecules in their structures depending on the void size and the chemical nature of each group. The accommodated particles are kept inside the dendrimers by different forces including electrostatic and hydrophobic forces, formation of complexes, van der Waals, and hydrogen bonds [39].

**Figure 4:**
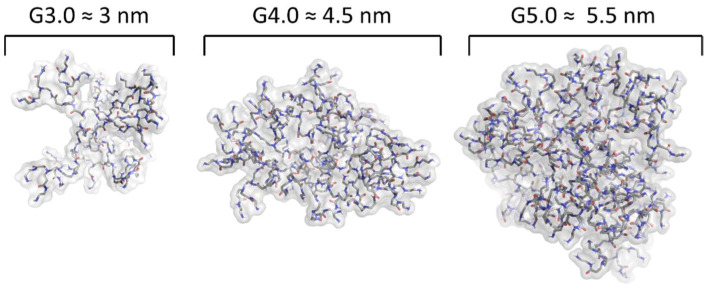
Molecular structure of PAMAM dendrimers of G3.0, G4.0 and G5.0 respectively, obtained after a DM of 100 ns through the force field OPLS3e.

The essential feature of dendrimers is their generation, defined as the number of layers attached to the core [40]. More layers attached to the core implies both a greater size and ionic charge. For instance, dendrimers of third generation poly(amidoamine) (PAMAM) (G3.0) have a diameter of 3 nm, similar to insulin, and fourth generation PAMAM (G4.0) have a diameter of 4 nm similar to cytochrome C. Fifth (G5.0) and sixth (G6.0) generations possess diameters equivalent to the thickness of lipid bilayer membranes of biological cells [40]. Figure 3 and Figure 4 show the structural arrangement of PAMAM dendrimers from G3.0 to G5.0 and how as they change generation, they increase their size and their amount of terminal amino groups in a relation of 2^(2+G)^, where G corresponds to the generation number for PAMAM dendrimers.

Modified dendrimers possess a high density of ionic charges on their surfaces, offering multiple attachment sites, which makes them attractive for the delivery of plasmid DNA, antisense oligonucleotides, and siRNA/dsRNA [41]. Moreover, the high density of charges contributes to the surface characteristics of the molecules and determines the molecular volume, which is important for the ability to separate other molecules within the dendrimer [34]. However, dendrimers that end in a cationic charge may present cytotoxicity and hemolytic properties [42] produced by an excess of positive charge, which can be toxic for cells, and this represents the major challenge in their clinical use. The dendrimers’ cytotoxicity may be associated with their size, charge and surface functionalization. The positive charge can react with the cell membrane, resulting in the creation of nanopores, subsequent leakage of cellular content, and eventually cell death. However, this can be reduced by protecting their charge through surface chemical modifications, such as functionalization with polyethylene glycol (PEG), pyrrolidone, acetyl groups, carbohydrates, and other moieties [40]. Bhadra et al. (2003) [43] found that PEGylation of G4.0 PAMAM dendrimers resulted in a significant reduction in the hemolytic and hematological toxicity of uncoated PAMAM dendrimers, with an improvement in drug loading capacity and a reduction in drug leakage.

Regarding clinical trials of dendrimers, in 2012, the formulation VivaGel^®^ was approved in Australia. This formulation consists of a dendrimer-based formulation that has been used for the treatment and prevention of sexually transmitted infections. This milestone underscores the immense potential of dendrimers as versatile drug delivery systems and therapeutic agents. The approval of VivaGel^®^ not only reflects the increasing interest in dendrimers as promising platforms for pharmaceutical development but also validates their potential to advance from technology to effective therapies for individuals [44,45,46].

### 2.1. Current Studies Using Dendrimers in TB

Research in the field of drug-loaded dendrimers for TB treatment has brought attention to the utilization of rifampicin and isoniazid as primary therapeutic agents. These two substances play a critical role in tuberculosis therapy and have garnered significant interest in studies aimed at enhancing their effectiveness and delivery via dendrimer encapsulation. Kumar et al. [47], Rajabnezhad et al. [48], and Ahmed et al. [49] reported on the prolonged release of rifampicin from poly(amidoamine) Pegylated dendrimers (PAMAM). Concurrently, Bellini et al. [50] demonstrated the remarkable stability of the rifampicin–PAMAM complex under physiological pH conditions and the rapid release of rifampicin in acidic environments, resembling the acidic niches within macrophages where *M. tuberculosis* resides. In addition, Rodrigues and Shende [51] employed the same PAMAM dendrimers for the delivery of isoniazid and copper. Furthermore, Mignani et al. [52] described the development of innovative, non-cytotoxic polycationic phosphorus dendrimers as potent anti-TB agents with inherent activity.

Table 1 provides a summary of research conducted on drug-loaded dendrimers for TB and their key findings.

### 2.2. Dendrimers for Gene Therapy

Dendrimers have proven to be effective gene delivery vectors, as they can form condensed complexes that are easily taken up by cells [54,55]. Commercial transfection reagents, such as Superfect and Priofect, can be used to create stable dendrimer/nucleic acid polyplexes. These complexes can buffer endosomes and prevent endolysosomal damage to nucleic acid through a proton sponge effect. The transfection and cytotoxic abilities of PAMAM dendrimers vary depending on their generation; lower generations (1–3) have lower transfection efficiency and cytotoxicity, while higher generations (4–8) have higher transfection efficiency and higher cytotoxicity, as the larger size and higher positive charge of the dendrimer can disrupt cell membranes and cause cellular damage [40]. Regarding cytotoxicity, it has been reported that dendrimers of a generation below G5.0 are less toxic than their higher-generation counterparts and adhere to genetic material with equal efficiency on their surface. The efficacy and effectiveness of genetic material delivery by PAMAM dendrimers have been well characterized in both in vitro and in vivo studies [56].

Furthermore, several studies have investigated the modification of dendrimers through PEG functionalization to enhance their hydrophilicity. This characteristic enables water molecules to establish hydrogen bonds with oxygen molecules within the PEG, resulting in the formation of a hydrated coating around the dendrimers. Consequently, this process aids in reducing the dendrimers’ immunogenicity and cytotoxicity [56,57].

Sharma et al. (2016) showed that PAMAM dendrimers of G1.0 are capable of delivering plasmids and siRNA in human cells, with an efficiency similar to Lipofectamine 2000, and proved that PAMAM dendrimers of G1 are less cytotoxic than Lipofectamine 2000 [54].

**Figure 5:**
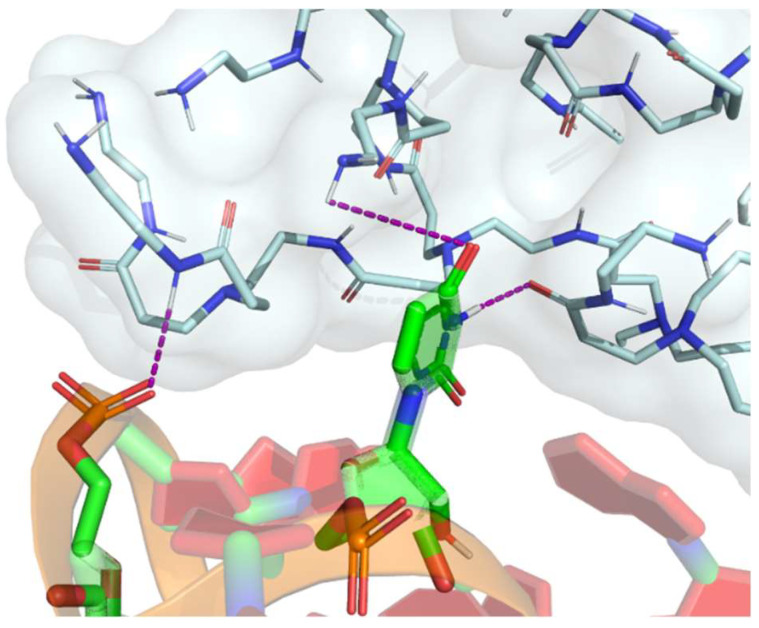
Exploration of intermolecular interactions between dendrimers and nucleic acids is illustrated in the diagram with purple lines indicating potential interactions between different functional groups present. For instance, one example is the interaction between the phosphate group of the nucleotide (in orange) and the amines of the dendrimer, or the interaction between the carbonyl group (in red) of the nitrogenous base and the hydrogen of the amine. Additionally, interactions can occur between a carbonyl group of the dendrimer and a nitrogen atom of the nitrogenous base. These interactions play a crucial role in understanding the binding and compatibility between dendrimers and nucleic acids, which can have implications in various applications such as drug delivery and gene therapy.

Polycationic dendrimers such as PAMAM and poly(propylenimine) (PPI) dendrimers have recently been used for RNA delivery. PAMAM dendrimers are widely employed for gene delivery because of their ease of synthesis and commercial availability [58]. The core of PAMAM is most commonly ethylenediamine, although more hydrophobic molecules—including diaminododecane, diaminohexane and diaminobutane—can also be used. Their branching units are based on methyl acrylate and ethylenediamine [42]. These have primary amine terminals, which are positively charged at physiological pH; thus, they can form stable complexes with negatively charged miRNA through electrostatic attractions with phosphate groups (Figure 5). Furthermore, as illustrated in Figure 5, PAMAM dendrimers demonstrate the capability to interact with phosphates, pentoses or nucleotides even when they are not linked to their nitrogenous base pairs. These efficient and biocompatible polymers could protect nucleic acids from degradation mediated by cytosolic nucleases [59]. Moreover, the complex dendrimers DNA or RNA are positively charged, which allows them to interact with negatively charged cell membranes and enter cells via endocytosis [60].

**Figure 6:**
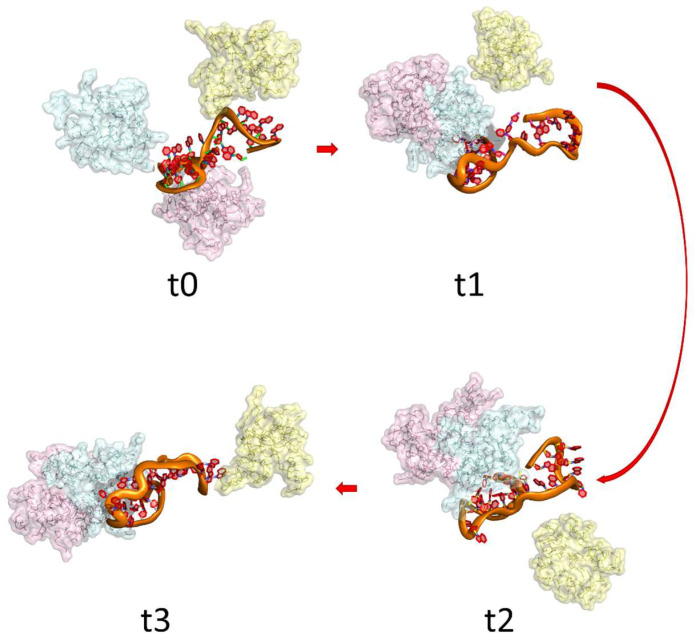
Visual representation of dynamic interactions, depicting the evolving interaction dynamics between three G3.0 PAMAM dendrimer molecules and miRNA over a dynamic simulation of 100 ns. t0 to t3 are different times during the molecular dynamic between miRNA and dendrimers.

Inapagolla et al. (2010) [61] studied G4.0 PAMAM dendrimers for pulmonary delivery of methylprednisolone (MP). They observed that the daily intranasal administration in mice for 5 days of G4.0 PAMAM functionalized with MP at 5 mg/kg did not cause any observable nonspecific inflammatory reactions within the lung. On the other hand, Dong et al. (2011) [62] studied the therapeutic effect of calcitonin and insulin administration loaded into PAMAM dendrimers of different generations (from G0.0 to G3.0). They found that the drugs loaded into higher PAMAM generations produce a higher therapeutic effect, especially G3.0 PAMAM dendrimers, which effectively increased the pulmonary absorption of insulin and calcitonin without any pulmonary damage. Royo-Rubio et al. (2021) [63] developed PEGylated carbosilane dendrimers to deliver miRNA as therapy against HIV-1 infection. They found that miRNA loaded dendrimers deliver the miRNA into the target cells and significantly inhibit HIV-1 infection in human peripheral blood mononuclear cells. Figure 6 presents a mechanistic illustration of the interactions between a G3.0 PAMAM dendrimer and an miRNA sequence. As depicted, dendrimers exhibit the capacity to interact not only with the miRNA but also to engage in dendrimer–dendrimer interactions through the interaction of amines with carbonyl groups within the dendrimer’s branches.

The cellular uptake of dendrimers is a complex process that is influenced by factors such as their size, surface charge, and shape. Various pathways can be involved, including phagocytosis, adsorption endocytosis, pinocytosis, or clathrin- and caveolin-mediated endocytosis. Once inside the cell, dendrimers are often located in endosomes, which are membrane-bound compartments that play a crucial role in the sorting and trafficking of cellular molecules. To achieve their desired therapeutic effect, it is important that dendrimers are able to escape from the endosome and deliver their cargo to the cytosol. This process is referred to as endosomal escape, and it can be a major bottleneck for the effectiveness of dendrimer-based delivery systems [64]. One way that dendrimers can achieve endosomal escape is through disintegration and release of their cargo into the cytosol. This can occur spontaneously during dendrimer swelling, which is a process that can be influenced by factors such as pH, temperature, and ionic strength. Once the dendrimer has disintegrated, the nucleic acid cargo is released and can exert its biological effects within the cell.

One of the key advantages of PAMAM dendrimers is their aqueous solubility and biocompatibility, which make them well-suited for use in biological applications and to carry RNA, siRNA or miRNA [65]. A study conducted by Bohr et al. [66] in the field of chronic lung inflammation revealed that PAMAM dendrimers loaded with siRNA can enhance cellular uptake in macrophages and reduce the expression of TNF-α, as demonstrated in an in vivo study.

### 2.3. Targeting Dendrimers to Alveolar Macrophages

The targeting of alveolar macrophages is controlled by the physicochemical parameters of particles such as size, shape, and surface characteristics. Peptide and small molecule-targeting ligands can be attached to dendrimers to improve cell-specific targeting [67]. In this sense macrophages are characterized by high expression of receptors for mannose, PPI, glycine, and tuftsin on the cell membrane. Therefore, our focus lies on functionalizing dendrimers with mannose and tuftsin, both of which are depicted below.

#### 2.3.1. Mannose Functionalization

Mannose is a common monosaccharide presented on pathogens’ surface and is commonly used to functionalize nanoparticles to target them to alveolar macrophages. Mannose receptor is involved in the recognition of pathogens, and in antigen processing and presentation [68]. The cell membrane mannose receptor is one of several types of recognition receptors that have evolved to exploit some of the essential surface structural features of related, common pathogens, such as the mannose-containing proteins or internal collagen sequences [69]. Mannose can direct proteins and peptides to macrophages in a receptor-mediated fashion, with resulting cellular activation. Thus, dendrimer functionalization with mannose may be used as a target to aim dendrimers to macrophages and release miRNA within the cell of interest.

Research conducted by Costa et al. (2018) [68] studied mannose-functionalized solid lipid nanoparticles loaded with isoniazid, an anti-tuberculosis agent, for targeting macrophages in vitro. They observed a much higher cellular uptake of isoniazid from the mannose-functionalized solid lipid nanoparticles compared to the non-functionalized nanoparticles in macrophages.

Sharma et al. (2021) [70] developed hydroxyl-terminated PAMAM dendrimers functionalized with different sugars (mannose, glucose and galactose) to target dendrimers to tumor-associated macrophages (TAM). The functionalization was developed through click reactions with sugar-azides modified with a short PEG linker to reduce steric hindrance to receptor interactions (β-d-Glucose-PEG4-azide, β-d-Galactose-PEG4-azide, and α-d-Mannose-PEG4-azide). The authors realized that the mannose- and glucose-functionalized dendrimers surprisingly produced the same TAMs, and microglia-targeted signal- and galactose-functionalized dendrimers exhibited highly distinct signal patterns due to the interaction with galectins which are highly overexpressed in different cancers. Compared with liposomal-functionalized structures, the glucose-functionalized dendrimers produced an internalization 100-fold higher than the non-functionalized dendrimers; meanwhile, the mannose- and galactose-functionalized dendrimers produced an internalization of 8-fold [70,71].

**Figure 7:**
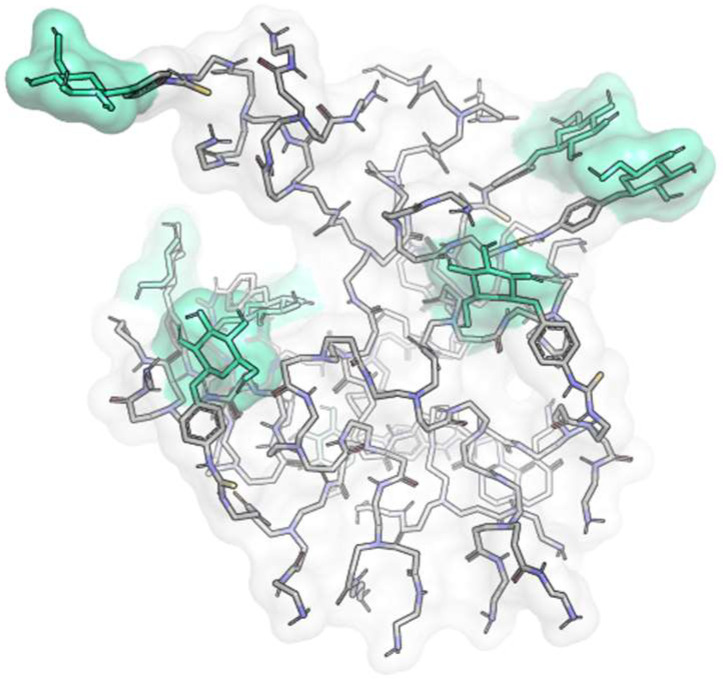
Molecular dynamic simulation of 100 ns depicting a mannose-functionalized G3.0 PAMAM structure.

In this review, we performed a dynamic simulation on a G3.0 PAMAM dendrimer functionalized with mannose to evaluate the structural conformation of PAMAM functionalized with mannose and the availability of mannose groups on the surface of G3.0 PAMAM. Figure 7 illustrates the tri-dimensional structure of the functionalized polymer.

#### 2.3.2. Tuftsin Functionalization

Tuftsin is a tetrapeptide that consists of L-threonine, L-lysine, L-proline, and L-arginine and which corresponds to the 289–292 amino acid sequence of the Fc portion of IgG [72]. This peptide has been shown to enhance the immune function of various cells, including macrophages, neutrophils, and monocytes via a specific receptor called CD11b/CD18. Tuftsin can increase macrophage-mediated phagocytosis, migration rate, splenocyte proliferation, and bactericidal and tumoricidal activities [73]. Furthermore, tuftsin binds to the transmembrane receptor neuropilin-1 (Nrp1), which is known to play critical roles in immunity and in cancer development [74]. Research has shown that there are approximately 72,000 binding sites for tuftsin on the surfaces of macrophages [75]. In a receptor-mediated manner, tuftsin can direct proteins and peptides to macrophages and activate the cells [76]. A number of different studies have been conducted using tuftsin as a targeting group, primarily in the field of liposome encapsulation to carry rifampicin or isoniazid, aiming to enhance macrophage uptake [72,77,78,79]. These studies have demonstrated that liposomes containing tuftsin are more efficiently internalized by macrophages compared with non-functionalized liposomes. Therefore, functionalizing dendrimers with tuftsin may serve to target dendrimers to macrophages and release miRNA within the cells of interest. Such an approach has the potential to revolutionize the treatment of diseases such as TB that rely on macrophage-mediated immune responses for successful treatment [72,80,81].

To react tuftsin with dendrimers, the amino group needs to be protected so that it does not react with the dendrimer’s functional groups before the desired reaction takes place. One common method of protecting the amino group is to use F_moc_ (9-fluorenylmethyloxycarbonyl) chemistry. This involves adding an F_moc_ group to the amino group, which temporarily blocks it from reacting with other molecules. The F_moc_ group can be removed later using a deprotection step, which frees up the amino group for further reaction with the dendrimer [75].

In 2008, Dutta et al. [75] conducted a study to investigate the potential of tuftsin-conjugated PPI dendrimers loaded with efavirenz, an antiretroviral drug, to target HIV-infected macrophages in vitro. The results showed a significantly higher uptake of efavirenz by HIV-infected fresh human mononuclear cells with tuftsin-conjugated dendrimers, compared with the free drug.

Another study reported on the functionalization of dendrimers with tuftsin for targeted delivery of siRNA to macrophages. The study demonstrated that the tuftsin-functionalized dendrimers exhibited improved uptake and silencing efficiency in macrophages compared with non-functionalized dendrimers.

However, to date, no studies have been conducted on the use of functionalized dendrimers with tuftsin to target miRNA for potential treatment of TB in macrophages. Given the challenges associated with targeted drug delivery to specific cell types, further research is necessary to determine the feasibility of this approach. Nonetheless, investigating new therapeutic strategies for TB remains critical, as it continues to be a significant public health concern.

## 3. General Outlooks and Future Perspectives

**Figure 8:**
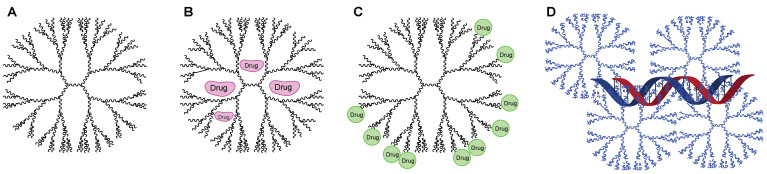
Strategy for the use of dendrimers. (**A**) Structure of the dendrimer. (**B**) Drug encapsulation within hydrophobic cavities of the dendrimer. (**C**) Drugs covalently bound in the polar terminal chains of the dendrimers. (**D**) Complex of dendrimers (usually named “dendriplexes”) with nucleic acid.

This paper investigated the potential role of dendrimers in TB treatment as delivery systems for miRNA-based therapeutic treatments (Figure 8). This ground-breaking strategy shows promise for boosting immune responses against *Mycobacterium tuberculosis* infection and avoiding the troubling problems posed by drug-resistant TB strains. By harnessing the unique attributes of dendrimers and designing them to mitigate cytotoxicity through different methods of functionalization or the addition of low molecular weight polymers, this innovative approach aspires to usher in a new era of sophisticated treatment paradigms, with the potential to be safely applied to individuals.

Central to the success of this approach is the functionalization of dendrimers with tailored ligands, such as mannose and tuftsin. These ligands, proficient in recognizing macrophage receptors, offer a precisely targeted delivery conduit that heightens therapy precision and efficiency [82]. This strategic ligand integration allows them to reinforce their capacity to engage with cells of pivotal relevance in the TB context.

However, to date, no studies have been conducted on the use of dendrimers functionalized with tuftsin or mannose to target miRNA for potential treatment of TB in macrophages. Given the challenges associated with targeted drug delivery to specific cell types, further research is necessary to determine the feasibility of this approach. Nonetheless, investigating new therapeutic strategies for TB remains critical, as it continues to be a significant public health concern.

In conclusion, investigating functionalized dendrimers targeted to macrophages as carriers for miRNA-based therapies represents a creative avenue in the search for groundbreaking TB treatment options. By designing these dendrimers to minimize cytotoxicity, we can enhance their safety for potential application in patients. This convergence of targeted medication delivery, immunology, and nanotechnology has the potential to fundamentally transform the landscape of TB intervention.

The development of sophisticated host-directed therapeutics utilizing dendrimers not only holds the promise of making TB treatment more effective but also more adaptable, addressing the challenges and uncertainties that currently exist in TB research. This innovative approach underscores the potential to trigger robust and adaptive immune responses, addressing pressing concerns related to drug resistance. It offers hope and the potential for success in the ongoing battle against tuberculosis.

## Figures and Tables

**Table 1:** Ongoing research investigates the utilization of dendrimers for encapsulating antibiotics in the treatment of tuberculosis (TB).

Anti-TB	Target	Polymers	Generation	Key Findings	Ref.
Rifampicin	Vero cells (ATCC-CCL-81e)	ethylene diamine, acrylonitrile (mannosylated dendrimer)	G5.0	The rifampicin-loaded mannosylated dendrimer reduced the drug release rate at pH 7.4, hemolytic toxicity, and cytotoxicity. Conversely, increased drug release at pH 5.0 and alveolar macrophages absorption were observed.	[47]
Rifampicin	-	Poly(amidoamine) PAMAM	G4.0	The results demonstrated the remarkable stability of the rifampicin–PAMAM complex at physiological pH, as well as the rapid release of rifampicin molecules in an acidic environment. This release pattern closely resembles the acidic domains found within macrophages, which are the host cells where *Mycobacterium tuberculosis* resides.	[50]
Rifampicin	Wister rats (male)—in vivo pulmonary drug absorption	Poly(amidoamine) PAMAM	G1.0-G3.0	The lower generation PAMAM microspheres were found to have a significant impact on the pharmacokinetic parameters of rifampicin, ultimately affecting the bioavailability of the drug. This study identified PAMAM G3 dendritic microspheres as suitable carriers for the pulmonary delivery of rifampicin to lung tissues.	[48]
Isoniazid and copper	*M. tuberculosis* H37Ra (ATCC25177) cells	Poly(amidoamine) PAMAM Methylmethacrylate	G4.0	The combination of copper and isoniazid showed a synergistic effect against *M. tuberculosis* H37Ra, resulting in a high inhibition rate of 96% and a significant dose reduction of up to 85 μg/mL. Copper nanoclusters containing isoniazid, synthesized using G4 PAMAM dendrimers, exhibited a controlled release profile with a cumulative drug release of 75% over 24 h.	[51]
Rifampicin	Raw 264.7 macrophage cells	Poly(amidoamine) PAMAM Poly(ethylene glycol) (PEG)	G4.0	The PEGylated G4 PAMAM dendrimers developed in this study are proposed as an ideal drug carrier for rifampicin, offering minimal cytotoxicity, high loading capacity, and extendedrelease characteristics.	[49]
Active per se	*M. tuberculosis* H37Ra *M. tuberculosis* H37Rv *M. bovis* BCG Balb/C mice	Polycationic phosphorus	G0.0–G4.0	The 2G0_HCl_ polycationic phosphorus dendrimer, has shown promise for treating TB based on in vitro and in vitro studies. It is a safe and chemically stable compound that remains intact in aerated aqueous solutions for up to 9 months, which is crucial for its potential use in clinical development. Notably, 2G0_HCl_ exhibits impressive efficacy against single drug-resistant strains of *M. tuberculosis* H37Rv that are resistant to rifampicin, isoniazid, ethambutol, or streptomycin. In vivo experiments using infected Balb/C mice have demonstrated significant effectiveness in reducing bacterial counts in the lungs when administered orally at a dose of 50 mg/kg once a day for 2 weeks, surpassing the efficacy of ethambutol and rifampicin.	[52]
Active per se	*M. tuberculosis* H37Rv	Glycoconjugated amine-terminated poly(ether imine) (PETIM)	G0.0 to G3.0	The Glyco-conjugated dendrimers PETIM possess antibacterial activity against *M. tuberculosis* by inhibiting its growth. The selectivity of the dendrimers towards mycobacterial growth inhibition was attributed to their glycosil moieties. The MIC values for *M. tuberculosis* H37Rv were found to be 100 mg/mL and 200 mg/mL, for both glycoconjugated G1.0 and G2.0, respectively.	[53]

## Data Availability

The data is currently not accessible to the public.
